# Understanding reproductive allometry in turtles: A slippery “slope”

**DOI:** 10.1002/ece3.5697

**Published:** 2019-10-02

**Authors:** John B. Iverson, Peter V. Lindeman, Jeffrey E. Lovich

**Affiliations:** ^1^ Department of Biology Earlham College Richmond IN USA; ^2^ Department of Biology and Health Sciences Edinboro University of Pennsylvania Edinboro PA USA; ^3^ U.S. Geological Survey Southwest Biological Science Center Flagstaff AZ USA

**Keywords:** clutch mass regression, clutch size, egg mass, log–log plot, pelvic aperture, scaling

## Abstract

Measures of reproductive output in turtles are generally positively correlated with female body size. However, a full understanding of reproductive allometry in turtles requires logarithmic transformation of reproductive and body size variables prior to regression analyses. This allows for slope comparisons with expected linear or cubic relationships for linear to linear and linear to volumetric variables, respectively. We compiled scaling data using this approach from published and unpublished turtle studies (46 populations of 25 species from eight families) to quantify patterns among taxa. Our results suggest that for log–log comparisons of clutch size, egg width, egg mass, clutch mass, and pelvic aperture width to shell length, all scale hypoallometrically despite theoretical predictions of isometry. Clutch size generally scaled at ~1.7 to 2.0 (compared to an isometric expectation of 3.0), egg width at ~0.5 (compared to an expectation of 1.0), egg mass at ~1.1 to 1.3 (3.0), clutch mass at ~2.5 to 2.8 (3.0), and pelvic aperture width at 0.8–0.9 (1.0). We also found preliminary evidence that scaling may differ across years and clutches even in the same population, as well as across populations of the same species. Future investigators should aspire to collect data on all these reproductive parameters and to report log–log allometric analyses to test our preliminary conclusions regarding reproductive allometry in turtles.

## Graphical abstract

Log–log scaling of reproductive variables against carapace length was examined for 46 turtle populations representing 25 species. Clutch size generally scaled at ~1.7 to 2.0 (compared to a theoretical isometric expectation of 3.0), egg width at ~0.5 (compared to an expectation of 1.0), egg mass at ~1.1 to 1.3 (3.0), clutch mass at ~2.5 to 2.8 (3.0), and pelvic aperture width at 0.8 to 0.9 (1.0). This approach is essential for understanding variation in reproductive output among turtles as well as any other taxon.

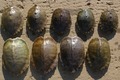

## 1 INTRODUCTION

**Figure 1:**
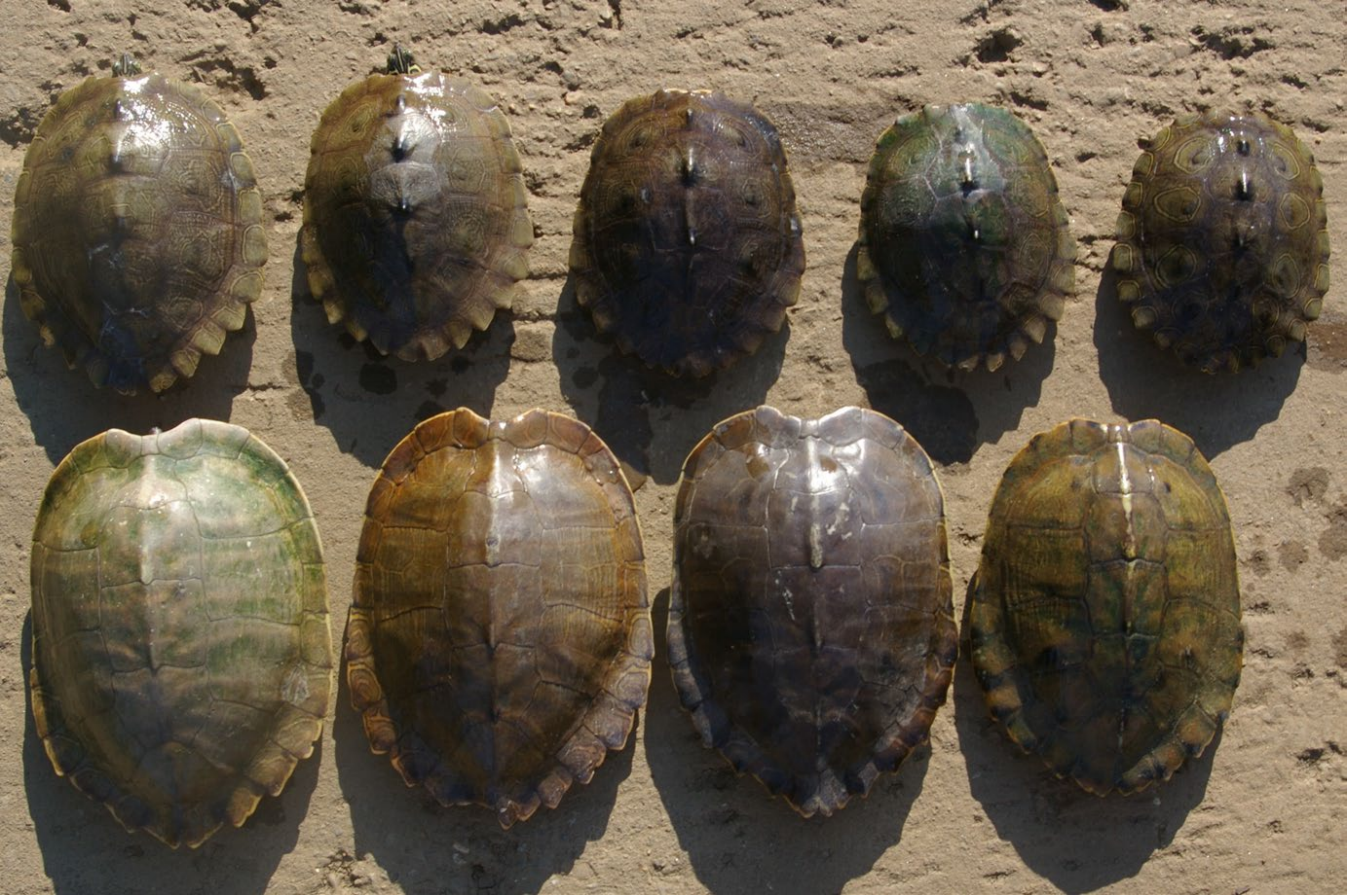
A series of gravid female turtles from the Alabama River in Autauga County, Alabama. The *Graptemys nigrinoda* in the upper row range from 145 to 177 mm in plastron length and the *Graptemys pulchra* in the lower row range from 185 to 207 mm in plastron length (see also Table 1)

Allometry, the study of the mathematical relationship of body size to life‐history traits, has proven to be a useful tool in quantifying and comparing the scaling of traits with body size both within and across species (Figure 1). Although most often applied to morphological and physiological traits, studies of the allometry of reproductive traits have been essential in understanding the evolution of reproductive strategies in organisms as diverse as plants (Hendriks & Mulder, 2008), crustaceans (Blueweiss et al., 1978; Hines, 1992), insects (Berrigan, 1991), fish (Blueweiss et al., 1978), salamanders (Kaplan & Salthe, 1979), reptiles (Hallmann & Griebler, 2018), lizards (Meiri, Brown, & Sibly, 2011; Warne & Charnov, 2008), and birds and mammals (Blueweiss et al., 1978). However, most of these studies of reproductive allometry have focused on interspecific comparisons, with very few focusing on these patterns within species.

Increases in measures of reproductive output with female body size are a common and expected pattern for most turtle species (Miller & Dinkelacker, 2008; Wilkinson & Gibbons, 2005). However, as pointed out by King (2000), comparative understanding of these patterns is enhanced if both body size and reproductive parameters are log‐transformed prior to subjecting them to linear regression analysis. This transformation reduces heteroscedasticity of variances and corrects for potential curvilinear relationships (King, 2000). It also allows for ease of comparisons of allometric changes in reproductive output among species, because it reduces the complications due to interspecific differences in body size.

The slope of log‐transformed reproductive data regressed against linear measurements of body size (e.g., carapace length or plastron length) is expected to be near 3.0 under isometry if the reproductive parameter is three‐dimensional (i.e., volumetric) in nature (e.g., egg mass, clutch size, or clutch mass), but if the reproductive parameter is linear (e.g., egg length, egg width, or X‐rayed egg width), the slope under isometry is expected to be near 1.0. If body mass is the measurement of body size, isometric slopes would be near 1.0 for three‐dimensional reproductive parameters and 0.33 for linear parameters.

Deviation from these expected values offers important and otherwise overlooked information on reproductive strategies among populations or species (King, 2000; Ryan & Lindeman, 2007). Under the hypothesis that selection has optimized egg size (Brockelman, 1975; Smith & Fretwell, 1974), an isometric relationship of clutch size to shell length (a measure of body size) is expected, with no significant relationship of egg size (e.g., mean egg width, mean egg length, or mean egg mass) to female shell length. Under an alternative hypothesis of pelvic or shell anatomy constraining egg size in smaller females (Congdon & Gibbons, 1987; Tucker, Funk, & Paukstis, 1978), both clutch size and egg size are expected to increase with female shell length, but they “compete” for the increased capacity for reproductive output and must both scale hypoallometrically with female shell length (Lindeman, 2005, Lindeman 2019; Macip‐Ríos, Brauer‐Robleda, Casas‐Andreu, Arias‐Cisneros, & Sustaita‐Rodríguez, 2012; Naimi, Znari, Lovich, Feddadi, & Baamrane, 2012; Ryan & Lindeman, 2007). We argue that because clutch mass (CM) is the product of mean egg mass (EM) and clutch size (CS), the log–log slope of CM regressed on shell length should equal the sum of the log–log slopes of CS and EM each regressed on shell length. If CS and EM both scaled with a slope of 3.0, CM would have to scale with shell length with an unrealistic slope of 6.0.

Turtle biologists have recently begun applying log–log regression techniques to within‐species reproductive studies (review in Lindeman, 2019), although not consistently enough for general allometric patterns to emerge. Clearly, an examination of these relationships based on actual field data from a diversity of turtle species is overdue. Therefore, we compiled published and unpublished log‐transformed allometric data for reproductive traits for 46 populations of turtles, representing 25 species and eight families, to test the prevailing predictions and potential patterns in intraspecific reproductive scaling in turtles. This study is the first to examine these patterns empirically within a diversity of turtles, although others have studied some of these patterns interspecifically (review in Hallmann & Griebler, 2018).

## 2 MATERIALS AND METHODS

### 2.1 Data collection

Slopes and associated 95% confidence intervals (CI) for body size–reproductive trait regressions were compiled primarily from the published literature. Body size (in mm) was measured as female carapace length (CL) or plastron length (PL), following the original authors. Reproductive traits included clutch size (CS), mean egg width per clutch (EW, in mm; from X‐rays in most cases but deposited clutches in a few cases), mean egg mass per clutch (EM, in g), clutch mass (CM, in g), and pelvic aperture width (PAW, in mm). These data were supplemented with our own unpublished data as well as those solicited from generous colleagues. Raw measurements of body size and reproductive output were log_10_‐transformed and then submitted to ordinary least‐squares linear regression analysis (OLS). Although it has been argued that reduced major axis regression (RMA) is more appropriate than OLS for calculating allometric slopes (Arnold & Green, 2007; LaBarbera, 1989; but see Warne & Charnov, 2008), no intraspecific study of turtle log–log allometry has used RMA. Hence, we were constrained in our analyses by the lack of data based on that approach.

Slopes with CIs that overlapped the expected slope of 1 or 3 were considered isometric while confidence intervals that fell outside the expected slope CI were considered either hypo‐ or hyperallometric (e.g., Arnold & Green, 2007). We also examined the consistency of slope values for populations with (a) different body size measurements (e.g., CL vs. PL), (b) data from different years, and (c) data for first versus second clutches in a given year, and (d) geographic variation in slopes from one species (*Sternotherus odoratus*) with data from four states. Finally, we examined the scaling data across habitat types by ANOVA, including terrestrial (*Terrapene*, *Chersina*, *Gopherus,* and *Homopus*), semiaquatic (*Kinosternon* and *Clemmys*), and aquatic taxa (all others).

For comparisons across taxa, the data set is inherently biased in that the number of samples is not constant across taxa (up to a maximum of seven for *Chrysemys picta*). The data are also obviously geographically biased toward North America. Initial analyses were nevertheless done with all samples, but repeated by averaging the slopes of all statistically significant slopes across samples for each taxon with multiple samples. We recognize the limitations of both of these methods, but given the relatively small data set available to us, our preliminary results should still provide insight to guide future work.

## 3 RESULTS

Our compiled data set included 46 populations of 25 species representing eight chelonian families (Table 1). No data were available from the diverse families Chelidae and Pelomedusidae or the monotypic families Carettochelyidae, Dermochelyidae, Dermatemydidae, and Platysternidae.

**Table 1:** Allometric comparisons of reproductive output with body size in turtles

Species	Location	CS	EW	EM	CM	PAW	Reference
Pleurodira							
Podocnemididae							
*Podocnemis expansa* (CL)	BR	**2.30** (30) 0.96–3.63	0.16^a^ (30) NC	1.01 (30) 0.24–1.79	4.55 (29) 3.15–5.95	–	T. C. G. Portelinha, A. Malvasio, C. I. Piña, and J. Bertoluci (2013, pers. comm.)
*Podocnemis unifilis* (CL)	VZ	0.40 (1,361) NR	–	0.06 (1,245) NC	–	–	Escalona et al. (2018)
Cryptodira							
Cheloniidae							
*Caretta caretta* (CL)	BR	**2.62** (27) 0.67–4.58	−0.11^a^ (28) NC	–	**2.43** (27) 0.30–4.56	–	M. Tiwari and K. A. Bjorndal (2000, pers. comm.)
*C. caretta* (CL)	FL	2.11 (48) 1.39–2.82	−0.02^a^ (48) NC	–	2.01 (48) 1.20–2.82	–	M. Tiwari and K. A. Bjorndal (2000, pers. comm.)
Chelydridae							
*Chelydra serpentina* (CL)	NE	1.32 (199) 1.08–1.57	–	0.98 (200) 0.85–1.12	2.30 (196) 2.03–2.56	–	A. R. Hedrick et al. (2018, unpublished)
*C. serpentina* (PL)	NE	1.30 (199) 1.04–1.56	–	1.03 (200) 0.90–1.16	2.33 (196) 2.05–2.61	–	A. R. Hedrick et al. (2018, unpublished)
Kinosternidae							
*Kinosternon flavescens* (CL; 2015)	NE	**2.51** (247) 2.02–3.00	0.69 (247) 0.60–0.77	–	–	–	J. B. J. B. Iverson (unpublished)
*K. flavescens* (PL; 2015)	NE	**2.94** (247) 2.41–3.47	0.73 (247) 0.63–0.82	–	–	–	J. B. Iverson (unpublished)
*K. flavescens* (CL; 2013)	NE	1.76 (134) 1.02–2.50	0.60 (134) 0.46–0.74	–	–	**1.01** (132) 0.82–1.20	J. B. Iverson (unpublished)
*K. flavescens* (CL; 2004)	NE	1.75 (278) 1.40–2.10	0.57 (275) 0.50–0.64	–	–	0.89 (283) 0.80–0.98	J. B. Iverson (unpublished)
*K. flavescens* (CL; 1988)	NE	1.63 (196) 1.20–2.06	0.60 (188) 0.50–0.70	–	–	0.78 (189) 0.66–0.90	J. B. Iverson (unpublished)
*Kinosternon integrum* (PL*)*	MX	1.98 (57) 1.25–2.71	0.14^a^ (57) 0.03–0.25	– (52) NC	1.86 (52) 1.16–2.56	–	Macip‐Ríos et al. (2012)
*K. integrum* (CL)	MX	2.08 (17) NC	0.29^a^ (14) NC	0.97 (14) NC	**3.16** (14) 0.68–5.63	–	J. B. Iverson (1999, unpublished)
*Kinosternon sonoriense* (CL)	AZ	3.24 (26) NC	0.00 (26) NC	–	–	0.47 (26) 0.20–0.74	J.E. Lovich et al. (2012, unpublished)
*Sternotherus carinatus* (CL)	AR	**2.81** (16) 1.04–4.58	0.32^a^ (16) NC	**1.98** (11) 0.56–3.39	**4.35** (10) 2.26–6.45	**0.98** a (16) 0.75–1.22	J. B. Iverson (2002, unpublished)
*Sternotherus odoratus* (CL)	TX	–	**0.61** (8) 0.09–1.13	–	–	1.23 (8) NC	Lindeman (2019)
*S. odoratus* (CL)	AR	2.22 (51) 1.51–2.93	0.20^a^ (43) 0.07–0.33	0.56 (41) 0.14–0.99	**2.50** (41) 1.63–3.37	–	J. B. Iverson (unpublished)
*S. odoratus* (CL)	FL	**3.87** (18) 2.51–5.23	−0.04 (10) NC	−0.36 (9) NC	4.47 (9) 3.33–5.60	–	J. B. Iverson (unpublished)
*S. odoratus* (CL)	IN	**2.30** (36) 1.10–3.51	0.54^a^ (24) 0.31–0.77	0.65 (12) NC	**3.79** (24) 2.36–5.22	–	J. B. Iverson (unpublished)
Emydidae							
*Chrysemys picta* (CL)	IL	1.38 (92) 0.59–2.17	0.42^a^ (73) 0.26–0.60	1.17 (94) 0.74–1.61	**2.61** (92) 1.84–3.37	–	Tucker in Ryan and Lindeman (2007)
*C. picta* (CL; 2012)	NE	0.82 (61) NC	–	1.99 (59) 1.26–2.72	**2.71** (58) 1.41–4.01	–	J. B. Iverson (unpublished)
*C. picta* (CL; 2012 first clutch)	NE	1.01 (21) NC	–	**2.32** (21) 0.80–3.84	**3.33** (21) 1.02–5.63	–	J. B. Iverson (unpublished)
*C. picta* (CL; 2013)	NE	2.06 (93) 1.35–2.77	–	1.77 (90) 1.26–2.28	3.85 (90) 3.20–4.50	–	J. B. Iverson (unpublished)
*C. picta* (PL; 2013)	NE	1.96 (93) 1.33–2.60	–	1.57 (90) 1.10–2.03	**3.54** (90) 2.96–4.12	–	J. B. Iverson (unpublished)
*C. picta* (CL; 2013 first clutch)	NE	1.94 (65) 1.11–2.78	–	1.89 (64) 1.28–2.50	3.84 (64) 3.07–4.61	–	J. B. Iverson (unpublished)
*Chrysemys* *picta* (CL; 1988–89)	NE	0.78 (100) 0.06–1.49	0.57^a^ (100) 0.43–0.70	1.70 (98) 1.29–2.12	**2.48** (98) 1.78–3.18	0.57 (100) 0.41–0.73	J. B. Iverson and G. R. Smith (1993, unpublished)
*Clemmys guttata* (CL)	CAN	1.49 (30) NC	0.34^a^ (26) 0.02–0.66	–	**2.42** (25) 0.21–4.60	**0.70** (29) 0.20–1.19	M. L. Rasmussen and J. D. Litzgus (2010, pers. comm.)
*Graptemys geographica* (PL)	PA	1.41 (50) 0.32–2.49	0.62 (43) 0.36–0.87	1.49 (17) 0.46–2.53	**2.66** (17) 1.04–4.27	**0.74** (26) 0.38–1.10	Ryan and Lindeman (2007), P. V. Lindeman (unpublished)
*Graptemys nigrinoda* (CL)	AL	**2.67** (31) 1.12–4.21	0.20 (31) NC	–	–	0.61 (31) 0.29–0.92	P. V. Lindeman (unpublished)
*Graptemys pulchra* (CL)	AL	**2.98** (11) −0.03–5.99	0.11 (11) NC	–	–	0.45 (11) NC	P. V. Lindeman (unpublished)
*Graptemys versa* (PL)	TX	2.05 (14) 1.13–2.97	0.44 (10) 0.24–0.64	–	–	**0.80** (10) 0.52–1.08	P. V. Lindeman (2005, unpublished)
*Graptemys sabinensis* (PL)	LA	0.92 (23) NC	0.28 (23) 0.10–0.47	–	–	**0.75** (23) 0.42–1.09	Fehrenbach et al. (2016)
*Malaclemys terrapin* (PL)	SC	–	0.45 (27) NR	–	–	**1.55** (20) 0.06–3.03	Kern, Guzy, Lovich, Gibbons, and Dorcas (2015)
*M. terrapin* (PL)	SC	–	0.22 (22) NR	–	–	**1.47** (65) 0.05–2.88	Kern et al. (2015)
*Terrapene ornata* (CL)	NE	1.53 (73) 0.24–2.82	0.17^a^ (29) NC	−0.05 (29) NC	1.37 (29) 0.13–2.61	0.25 (20) NC	J. B. Iverson (unpublished)
*Trachemys scripta* (CL)	IL	1.70 (4,463) 1.59–1.81	0.45^a^ (2,763) 0.42–0.47	1.20 (4,667) 1.14–1.25	**2.89** (4,458) 2.78–3.00	–	Tucker, in Ryan and Lindeman (2007)
*T. scripta* (CL)	AR	1.40 (17) NC	–	0.57 (17) NC	**1.98** (17) 0.35–3.60	–	J. B. Iverson (unpublished)
Geoemydidae							
*Mauremys leprosa* (CL)	MR	2.03 (28) 1.61–2.45	0.74^a^ (28) 0.56–0.91	0.71 (28) 0.46–0.95	**2.73** (28) 2.30–3.17	1.43^a^ (28) 1.24–1.62	Naimi et al. (2012)
Testudinidae							
*Chersina angulata* (CL)	CA	–	–	0.98 (25) 0.14–1.82	–	–	M. D. Hofmyer (unpublished)
*Gopherus agassizii* (CL; first clutch)	CA	**1.67** (64) 0.19–3.16	–	–	–	–	J. E. Lovich et al. (2015, in part)
*G. agassizii* (CL; first clutch)	CA	1.43 (19) NC^b^	0.26 (19) NR	–	–	–	Lovich et al. (2018)
*G. agassizii* (CL; second clutch)	CA	−0.24 (52) NC	–	–	–	–	J. E. Lovich et al. (2015, in part)
*G. agassizii* (CL; second clutch)	CA	2.62 (14) NC	0.00 (14) NC	–	–	–	Lovich et al. (2018)
*Gopherus polyphemus* (PL)	FL	1.58 (27) NR	0.49 (26) NR	–	–	**0.95** (26) NR	Rothermel and Castellón (2014)
*Homopus signatus* (CL)	AF	–	0.69 (31) NR	–	–	0.70 (31) NR	Hofmeyr et al. (2005)
Trionychidae							
*Apalone ferox* (PL)	FL	1.79 (45) 1.18–2.39	0.09^a^ (41) NC	0.32 (14) NC	**2.56** (14) 1.77–3.54	–	J. B. Iverson et al., 1997, unpublished)

Note

Reproductive traits are clutch size (CS), egg width (EW, by X‐ray or actual measurement), mean clutch egg mass (EM), clutch mass (CM), and pelvis aperture width (PAW, by X‐ray or actual measurement). Body size trait (carapace length, CL, or plastron length, PL) appears after the species name. Slope (and sample size in parentheses) appears above the 95% confidence intervals of the slope. Slopes in boldface include the slope of 3.0 or 1.0 that is consistent with isometry within their confidence intervals.

NC, not correlated (*p* > .05); NR, not reported.

AF, Africa; BR, Brazil; CAN, Canada; MR, Morocco; MX, Mexico; VZ, Venezuela; others are states in the USA.

a Based on direct measurement of egg or pelvic width rather than from X‐rays.

b Note that if single egg clutches were removed from this data set, the slope (2.70) was statistically significant.

### 3.1 Scaling of reproductive traits

#### 3.1.1 Pelvic aperture width

As expected, PAW was generally correlated with shell length, except in three small samples: *Sternotherus odoratus* (*N* = 8 individuals), *Graptemys pulchra* (*N* = 11), and *Terrapene ornata* (*N* = 20). PAW nearly consistently scaled to shell length with a slope of 1.0 or less (mean of 16 statistically significant slopes in Table 1 = 0.90; median = 0.79). Nine of 14 samples (64%; eight species) for which confidence intervals were available included 1.0, consistent with isometry. However, the single studied population of *Mauremys leprosa* demonstrated hyperallometry in actual pelvic aperture width (slope = 1.43; Naimi et al., 2012). Similarly, although not significantly different from 1.0, the slopes for two populations of *Malaclemys terrapin* were 1.47 and 1.55. These values for *Mauremys* and *Malaclemys* were the only values that exceeded 1.00 (Table 1). Exclusion of these latter two values from the analysis yielded a mean slope of only 0.81 for the remainder of our sample. In contrast, populations of *C. picta* in Nebraska (two years' data; slope = 0.57), *K. sonoriense* in Arizona (0.47), and *Graptemys nigrinoda* in Alabama (0.61), exhibited significant hypoallometry in spite of large sample sizes (*N* = 100, 26, and 31 individuals, respectively). Overall, the available data suggest that pelvic aperture width typically scales with slight hypoallometry in turtles, with a modal range of slopes of 0.80–0.90. Indeed, the confidence intervals of 11 of the 14 available samples included 0.85, two more than included 1.0.

#### 3.1.2 Clutch mass

Every available CM sample (*N* = 25 samples) demonstrated a significant positive relationship between shell length and CM (Table 1). The mean slope for statistically significant regressions of log‐transformed clutch mass on shell length for those 25 populations (representing 13 species) was 2.91 (median = 2.66), similar to the isometric slope of 3.0, and of the 25 samples for which confidence intervals were available, the intervals for 15 (60%) included 3.0. However, slopes for individual samples varied widely. As examples, significant slopes for samples of *S. odoratus* ranged from 2.50 to 4.47 (*N* = 3 samples), those for *Kinosternon integrum* from 1.86 to 3.16 (*N* = 2), and those for *C. picta* from 2.61 to 3.65 (*N* = 7). Furthermore, for only those 12 samples (six species) that had *N* > 30 individuals, the mean slope was 2.75, although that mean was inflated by two samples of *C. picta* (slopes 3.84 and 3.85; mean slope excluding those two = 2.52). And finally, we calculated the mean slope for each species, including only those with *N* > 30 individuals for multiple samples, and when averaged across the six species, the mean slope was 2.47. These data suggest that CM generally scales to shell length slightly hypoallometrically, with a modal slope range of 2.5–2.8. Furthermore, the CIs of 19 of the 25 available samples (76%) included 2.5, four more than included 3.0.

#### 3.1.3 Clutch size

Clutch size correlated positively with shell length in most samples (31 of 41 [76%], including at least one population for 19 of 22 species [86%]; Table 1). Considering only those samples with *N* > 30 individuals, CS was correlated with shell length in 23 of 26 (88%). The mean slope for statistically significant log‐transformed regressions of CS on shell length for 31 populations of 19 species was 1.98; median = 1.96, far below the isometric slope of 3.0 (i.e., strongly hypoallometric). Slopes for CS also varied widely, from hypoallometric (0.40 for *Podocnemis unifilis*, and 0.78 for two years' data from one population of *C. picta*) to hyperallometric (3.87 in one population of *S. odoratus*). Only nine of 29 samples (seven species) had confidence intervals including 3.0, although sample sizes were ≥ 18 for only four of the seven species. For only those 25 significant samples with *N* > 30 individuals (14 species), the mean slope was 1.83. Finally, we calculated the mean slope for each species with multiple samples, including only those with *N* > 30, and when averaged across the 14 species, the mean was 1.72. For most turtles, CS scales hypoallometrically with shell length, with modal values of 1.7–2.0. Significantly, the CIs of 22 of the 29 available samples (76%) included 1.85, 13 more than included 3.0.

#### 3.1.4 Mean egg mass

**Figure 2:**
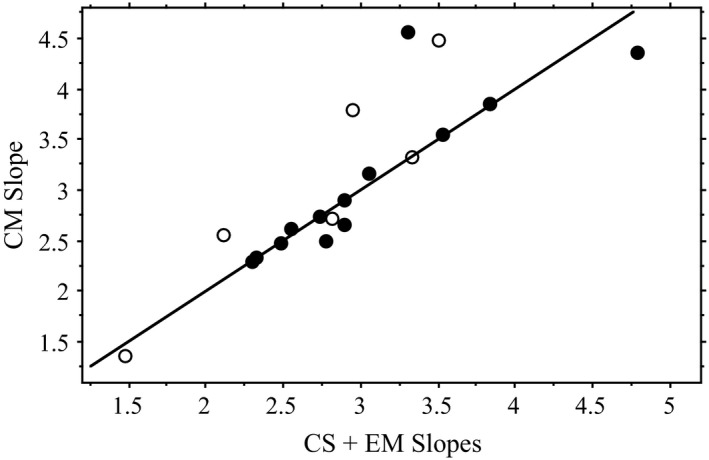
Relationship between the sum of the log–log slopes of clutch size (CS) and egg mass (EM) regressed on shell length and the log–log slope for clutch mass (CM) regressed on shell length. The line indicates the expected 1:1 relationship among these slopes (CS + EM = CM). Filled symbols represent cases in which all three relationships with shell length were significant, while open symbols represent cases having one or more nonsignificant relationships

Egg mass correlated positively with shell length in 16 of 24 samples (67%, including at least one population among ten species; Table 1). Considering only those 12 samples with *N* > 30 individuals, EM was correlated with shell length in ten (five species). The mean slope for the statistically significant log‐transformed regressions of EM on shell length for 16 populations of ten species was 1.40 (median 1.35), less than half the isometric slope of 3.0 (extreme hypoallometry). One population of *S. odoratus* (0.56) and *M. leprosa* (0.71) exhibited the most extreme hypoallometry, and one population of *C. picta* had the highest (least) hypoallometry (2.32). The only two populations with CIs including 3.0 had sample sizes of only 11 and 21 individuals. When we calculated a mean slope for each species with multiple samples, each with *N* > 30 individuals, the average slope across the four species was 1.11. EM did not scale to shell length with the expected slope of 3.0 in turtles, but rather exhibited hypoallometry with a typical slope of 1.1–1.3. Furthermore, the CIs of 8 of the 16 available samples (50%) included 1.2, six more than included 3.0. As expected mathematically, the sum of the significant slopes for CS and EM approximated that of CM within species and populations (Figure 2).

#### 3.1.5 Mean egg width

Egg width (from either X‐rays or oviposited eggs) correlated positively with shell length in 22 of 34 samples (65%, including at least one population for 14 species; Table 1). Considering only those 14 samples with *N* > 30 individuals, EW was correlated with shell length in 12 (86%, seven species). The mean slope for the statistically significant log‐transformed regressions of EW on shell length for 22 populations of 14 species was 0.48 (median = 0.51), about half the expected isometric slope of 1.0 (extreme hypoallometry). The most hypoallometric samples were one population of *Kinosternon integrum* (slope = 0.14), one of *S. odoratus* (0.20), and one of *M. terrapin* (0.22), all with sample sizes of 22–57 individuals. The steepest slopes were found in *M. leprosa* (0.74) and one population of *K. flavescens* (0.73), with *N* = 28 and 247 individuals, respectively, but neither species' CIs included the value of 1.0 expected under isometry. Only one sample (one of four for *S. odoratus*), with a sample size of only eight, had a CI that included 1.0. For only those 12 significant samples with *N* > 30 individuals (seven species), the mean slope was 0.52. Finally, we calculated the mean slope for each species with multiple samples, including only those with *N* > 30 individuals, and when averaged across the seven species, the mean was 0.46. Egg width in turtles did not scale with shell length with the isometric slope of 1.0, but rather was consistently hypoallometric with a modal slope of 0.5. Strikingly, the CIs of 10 of the 17 available samples (59%) included 0.5, nine more than included 1.0.

### 3.2 Confounding morphological and life‐history factors

For three species (*C. serpentina* in Nebraska across many years; *C. picta* in Nebraska in 2013; and *K. flavescens* in Nebraska in 2015), slope data using both CL and PL were available to test the effect of the body size measurement on reproductive parameters. Based on their broadly overlapping CIs, method of shell measure apparently had little effect on the allometry of reproductive output.

For only one species (*K. flavescens*), slope data were available from the same population in Nebraska for four different years for several of the output parameters. No year effect was evident for PAW or EW based on overlapping CIs, but the slope for CS was greater in 2015 than in 1988, 2004, and 2013, based on the nonoverlap of the CI for 2015 with the means of the other three years. This suggests a possible annual effect.

For two species (*C. picta* and *Gopherus agassizii*), slope data were available across clutches during the nesting season. In *C. picta,* for the first clutch of the season versus the full season in either 2012 or 2013 in Nebraska, the slopes for CS, EM, and CM did not differ between the samples (based on broadly overlapping CIs). The sample size for first versus second clutches in *G. agassizii* was limited, but suggested that EW for first clutches was correlated with CL, but not for second clutches.

For one species (*S. odoratus*), data for some parameters were available from Florida, Texas, Arkansas, and Indiana. The slope for CS in Florida was significantly higher than those for the other states, based on the nonoverlap of the confidence interval for Florida with the means of the other three years. Slopes for EW and EM were similar among states.

For all five reproductive variables, we found no significant variation in slope values among terrestrial, semiaquatic, and aquatic species (*F* < 0.76 and *p* > 0.39 for all comparisons). For CM, only one terrestrial sample was available (slope = 1.37), precluding inclusion in an ANOVA. However, given the mean slope for semiaquatic species (2.48 ± 0.65) and that for aquatic taxa (2.95 ± 0.90), a weak pattern may exist for decreasing CM slope with increasing terrestriality.

## 4 DISCUSSION

Because shell length, PAW, and EW are one‐dimensional measurements, and CS, EM, and CM theoretically represent cubic, volumetric measurements (Froese, 2006), log–log regressions of PAW and EW to shell length would scale isometrically with a slope of 1.0, whereas those of CS, EM, and CM would scale isometrically with a slope of 3.0 (King, 2000; Ryan & Lindeman, 2007). All the reproductive variables we examined generally scaled hypoallometrically with shell length compared to the expectation of isometric log–log slopes. Clutch mass (modal slope = 2.5–2.8; predicted slope = 3.0) and pelvic aperture width (=0.80–0.90; predicted slope = 1.0) scaled most closely with predicted isometry, although clutch size (1.7–2.0; 3.0), egg width (0.5; 1.0), and egg mass (1.1–1.3; 3.0) scaled with significantly lower slopes. As expected mathematically, the sum of the significant slopes for CS and EM approximated that of CM within species (Figure 2).

Although this paper focuses on statistically significant log–log regressions of reproductive traits, it is also of interest to mention those studies that found no significant relationship (i.e., slopes not different from zero). Nine samples representing nine species with *N* > 28 individuals exhibited this condition, three involving CS and six involving egg size (Table 1). Of the former three, one probably reflects the small CS for the species (*Clemmys guttata*), one represents only second clutch data from two populations (*G. agassizii*), and one population of another species includes mixed seasonal clutches from the warmest year in 46 years at the site (*C. picta* in 2012; J. B. Iverson unpublished). Lack of correlation between CS and shell length in a turtle population is clearly unusual (e.g., Elgar & Heaphy, 1989). However, a lack of correlation between egg size and shell length is more common (12 of 34 cases involving egg width and 8 of 24 involving egg mass; Table 1). For those seven with large sample sizes in Table 1, six (*Caretta caretta*, *K. integrum*, *K. sonoriense*, *G. nigrinoda*, *T. ornata*, and *Apalone ferox*) had constant egg sizes across shell length, perhaps suggesting selection for an optimal egg size. The last case lumped multiple populations (*Podocnemis expansa*) and had a significant CL‐EM correlation, but no relationship between CL and EW. No sample lacked a significant positive correlation between shell length and CM.

### 4.1 Scaling of reproductive traits

#### 4.1.1 Pelvic aperture width

Although PAW scales with shell length close to isometry (i.e., a slope of 1.0), the typical pattern is still hypoallometric. This pattern may simply reflect an ontogenetic elongation of the shell relative to pelvic (i.e., body) width (Brophy, 2006; Ernst, Wilgenbusch, Boucher, & Sekscienski, 1998; Fish & Stayton, 2014; Froese, 2006; Kamazaki & Matsui, 1997), and/or adaptive changes in pelvic structure related to locomotion (Lovich et al., 2012). Because EW scales at only about half the slope as for PAW, the latter probably never constrains egg size in the large turtles of a population (Rollinson & Brooks, 2008). It should be noted that no author has verified the accuracy of measuring pelvic width from X‐rays compared with actual pelvis aperture width by dissecting radiographed museum specimens. However, Clark, Ewert, and Nelson (2001) and Naimi et al. (2012) are apparently the only authors to have measured actual PAW from dissected turtles. Whether measurement error from X‐rays might explain the observed deviations from isometry in some turtle populations is unclear.

#### 4.1.2 Clutch mass

That CM generally scales hypoallometrically with shell length in turtles was surprising. However, this pattern may be due in part to an ontogenetic faster increase in shell length relative to carapace width, carapace height, and thus abdominal volume. For example, Iverson (1984) found that log–log slopes of body mass regressed against CL for 39 turtle populations (representing 25 species) averaged 2.75 (i.e., hypoallometric, and similar to typical CL‐CM slopes of 2.5–2.8), suggesting that carapace length is not a reliable measure of actual body volume (see also Zuffi, Odetti, & Meozzi, 1999). Hence, volumetric measures in turtles might generally be expected to scale with shell length at a slope of 2.75, rather than 3.0. (i.e., hypoallometrically).

The hypoallometric slope for CM might also suggest that females may not be maximizing the use of the abdominal cavity for proximate reproductive output. Volumetric studies of the abdominal components of gravid turtles over a range of body sizes would be very informative (e.g., using magnetic resonance imaging). For example, we would predict that adipose tissue (fat bodies) might occupy a larger relative volume of the abdomen in larger turtles, perhaps limiting space for reproductive output (Georges, 1983; Kwan, 1994).

However, despite the trend for hypoallometry, four samples exhibited statistically significant hyperallometry (slopes > 3.0), which seems physically unlikely unless carapace height increases very significantly with shell length. One of those (*S. odoratus* in Florida) included only nine turtles, which may be a biased sample. The others (*P. expansa* and *C. picta* from 2013 in Nebraska) had large samples and cannot be easily explained. This hyperallometry in clutch mass in some turtles is unexpected and deserves further attention.

#### 4.1.3 Clutch size

Most turtle populations exhibit a positive correlation between shell length and clutch size (Elgar & Heaphy, 1989; Miller & Dinkelacker, 2008; Rasmussen & Litzgus, 2010; among many others), and as noted above, it is rare for a well‐sampled population not to demonstrate this relationship (e.g., Gibbons & Greene, 1990). Optimal egg size theory predicts that increases in reproductive output should manifest in increases in CS rather than egg size (Brockelman, 1975; Smith & Fretwell, 1974), and hence, this correlation is expected except perhaps in species with clutches of one or only a few eggs (Hofmeyr, Henen, & Loehr, 2005; Lindeman, 2019). However, CS consistently scales hypoallometrically with shell length. Only a single turtle sample, a small (*N* = 11 individuals), mixed‐location sample of *S. odoratus* from Florida, exhibited statistically significant hyperallometry and must be considered anomalous. Future allometric studies should not expect CS to scale with a theoretical slope of 3.0, but rather with values of 2.0 or less.

#### 4.1.4 Mean egg mass

Egg mass is correlated with shell length in most turtles, though not as frequently as for CS, as predicted by optimal egg size theory (Congdon & Gibbons, 1987; Ryan & Lindeman, 2007), and generally observed in nature (e.g., Elgar & Heaphy, 1989). However, like CS, EM does not scale to shell length with a slope of 3.0 when samples are adequate, and indeed typically scales at less than half that slope. Future studies should not expect the predicted slope but rather make comparisons with the lower slope demonstrated here. Authors should also expect the slope for CM to equal the sum of those for EM and CS (Figure 2). Furthermore, differences in egg size allometry should be expected across clutches within a season, as previously demonstrated by Iverson and Smith (1993), Doody, Georges, and Young (2003), and Ennen et al. (2017).

Previous work has suggested that hypoallometry of both egg and clutch size in turtles is a result of competing sinks for increased reproductive investment of larger (presumably older) females (i.e., the egg size‐clutch size trade‐off; reviewed by Lindeman, 2019). However, given that CM (the product of EM and CS) is also generally slightly hypoallometric in turtles, hypoallometry in both EM and CS is the only possible expectation, even if there is no clear trade‐off between the two. Hence, comparing the slopes of CS and EM relative to that of CM for a population should be much more informative to understanding patterns of reproductive output than simply making comparisons with theoretical slopes.

#### 4.1.5 Mean egg width

As for EM, allometry in EW is half as steep as predicted. Future work should strive to collect data relating EW to EM, permitting an examination of allometry in both variables. Furthermore, egg shape (i.e., egg volume or egg elongation) has often been overlooked in studies of reproductive allometry in turtles. This is a significant challenge in comparative studies like ours because some turtle species lay spherical eggs and others are ellipsoidal to various degrees (Iverson & Ewert, 1991). Although some authors have demonstrated positive correlations of both EL and EW with shell length (e.g., Iverson, Griffiths, Higgins, & Sirulnik, 1997; Iverson & Smith, 1993) or no correlation of either EL or EW with shell length (e.g., Iverson & Moler, 1997), others have only found a correlation of EW but not of EL with shell length (Lindeman, 2005; Macip‐Ríos et al., 2012; Naimi et al., 2012; Rasmussen & Litzgus, 2010; Ryan & Lindeman, 2007) and still others have found a correlation of EL but not EW with shell length (Escalona, Adams, & Valenzuela, 2018). This variation suggests that egg elongation (EE: EL/EW) may be an important feature of reproductive output, potentially allowing small turtles to overcome pelvic or caudal aperture constraints; however, only a few studies have attempted to quantify it and relate it to shell length. Four such studies found a negative correlation between EE and shell length (Clark et al., 2001; Escalona et al., 2018; Iverson & Moler, 1997; Macip‐Ríos et al., 2012), and three others found no relationship (Iverson et al., 1997; Iverson & Smith, 1993; Macip‐Ríos et al., 2012). However, only the study by Escalona et al. (2018) performed log–log transformations of the data prior to analysis. In any case, the possibility that at least some turtles are increasing egg size in small adults (and potentially avoiding pelvic aperture constraints) by producing relatively elongate eggs argues against relying solely on X‐ray egg widths as a measure of egg size without examining their relationships with egg length and egg mass.

### 4.2 Theoretical implications

Our results provide some evidence supporting OES theory, in that CS usually increases significantly with body size (28 of 35 populations with data). However, 16 of 24 populations with EM data showed a positive correlation with body size, contrary to the prediction of OES theory. Instead, these results combined with the general pattern of hypoallometry in reproductive traits in most turtles lend support for a bet‐hedging strategy (Olofsson, Ripa, & Jonzén, 2009). Turtles in general may not maximize their reproductive output in a given bout (hence, lowering their near‐term fitness) in order to increase (or maximize) their long‐term reproductive output (i.e., fitness). One test of the applicability of this theory to turtles would be to determine whether reproductive allometric slopes are higher for turtle populations in less variable and more predictable environments (Ennen et al., 2017).

### 4.3 Confounding factors

Considerable variation exists regarding the methods by which shell length is measured in turtles (Iverson & Lewis, 2018). However, our preliminary data suggest that either carapace length or plastron length is acceptable as a measure of body length for calculating and comparing scaling data. The two measurements likely are often very highly correlated within populations (but see Lovich, Ernst, & McBreen, 1990), so regression of reproductive output variables on either should give similar results. However, we did not examine scaling of reproductive traits using body mass (or other volumetric measures), primarily because very few authors report such data. Given the diversity in body shapes among turtle species (Pritchard, 2008) and the variation in shell shape (particularly, shell height and volume) among habitats within many species (Ennen et al., 2014; Rivera, Davis, Godwin, & Adams, 2014; Selman, 2012), future studies of reproductive allometry should aspire to incorporate body mass or body volume as more appropriate measures of body size than CL or PL. It is likely that variation in shell height will explain considerable variation in clutch size and mass across species.

Small sample sizes are more likely to have larger confidence intervals that include theoretical slopes, and hence may mislead authors about the frequency of isometry. Future studies should aim for sample sizes > 30 across the entire range of adult body sizes to minimize confidence intervals and reliably estimate log–log slopes. In addition, in the future the simultaneous collection of CS, CM, and especially EM data is sorely needed. Although radiographing gravid females is informative and much less time‐intensive than collecting reproductive data directly from nesting females, the latter are necessary for CM and EM data to allow more thorough analyses of reproductive output in turtles, especially when used in combination with radiographs.

We demonstrated considerable variation in scaling patterns among samples from the same species, presumably reflecting local adaptation and/or acclimation and/or measurement error. Hence, for future work, populations should not be lumped in studies of reproductive output in a given species. An extreme example of the danger of doing so is evident in figure 3 of Escalona et al. (2018), which suggests considerable hidden interpopulation variation in relative egg length (i.e., elongation) in their lumped sample. Furthermore, when possible, separating reproductive data by clutch number within a season may reveal important patterns of variation, especially given that numerous authors have demonstrated differences among seasonal clutches using nonscaled data (e.g., Doody et al., 2003; Ennen et al., 2017; Iverson & Smith, 1993; Tucker & Frazer, 1994).

The variation among the samples of *C. picta* and *K. flavescens* from the same Nebraska field site suggests that annual variation in climate (and associated impacts on resource availability) may be an important factor affecting reproductive allometry (see also Doody et al., 2003; Iverson & Smith, 1993). For example, 2012 was the warmest year at the Nebraska site in the 46 years of our records (Iverson, unpublished) and climate has been shown to impact both clutch and egg size (and hence clutch mass) in other turtles (Hedrick, Klondaris, Corichi, Dreslik, & Iverson, 2018; Iverson & Smith, 1993; Rollinson, Farmer, & Brooks, 2012). The effects of annual variation in climate on reproductive output in turtles remain poorly studied, and incorporating that variation in scaling studies will likely be rewarding. At the least, data from different years should not be lumped for analysis.

Our preliminary analyses of variation of reproductive scaling by habitat preference revealed only a weak possible pattern of decreased slope for CM with increasing terrestriality. However, no differences across habitat types were found in an earlier interspecific study (Iverson, 1992). Nevertheless, future work is needed to test for habitat (or other life history) effects on reproductive allometry in turtles.

Additional factors that might affect reproductive scaling are also worthy of attention (see also Lovich et al., 2012). For example, size‐adjusted CM in carnivorous species is generally greater than that in herbivorous species (Iverson, 1992; Jackson, 1988) and this pattern should be reflected in allometric analyses. Furthermore, clutch frequency within years has been shown to be inversely related to size‐adjusted CM across turtle species (Iverson, 1992) and may also explain some of the variation in slopes we observed.

Finally, future analyses of reproductive allometry in turtles should examine log–log regressions (especially of egg size) for inflection points (e.g., by broken stick modeling; see Toms & Lesperance, 2003) along the body size continuum. For example, DePari (1988), Rollinson and Brooks (2008), and Macip‐Ríos et al. (2012) identified such a pattern of inflection, with near‐constant egg size in larger turtles, without explicit modeling, in *C. picta* (two studies) and *K. integrum*, respectively. How common this pattern is among turtles remains to be determined but it may be that many turtle populations show the effects of anatomical constraints on egg size among smaller females but are able to optimize egg size among larger females (Lindeman, 2019).

### 4.4 Broader taxonomic applications

As outlined in the Introduction, interspecific log–log reproductive allometric analyses are commonly reported for plants and animals. On the contrary, such analyses within populations are only rarely reported beyond turtles (Table 2). Other than an early report including allometric slopes for six species of crabs (Hines, 1992), ours is the first study to attempt to compile such data for a large sample (46 populations) of a higher taxon. Unfortunately, the available data permit little speculation on reproductive allometric patterns beyond turtles. For example, of the two snakes with allometric clutch size data (Table 2), one apparently is isometric and the other decidedly hypoallometric. Future studies are urged to submit their data to log–log allometric analyses and to publish those data.

**Table 2:** Literature records for intraspecific analyses of reproductive allometry in animals

Taxon	Number species	Size	CS	EM	CV	CM	Source
Crabs	6	BM	1.15 (1) (0.70–0.91)	–	–	1.11 (1) (0.74–1.79)	Hines (1992)
Dusky Salamander	1	BL	1.78 (3)	–	1.76 (3)	–	Bruce (2014)
Ambystomatid Salamanders	4	BV	0.60 (1) (0.24–0.97)	–	0.88 (1) (0.55–1.15)	–	Kaplan and Salthe (1979)
Green Iguana	1	BL	2.97 (3)	–	–	–	King (2000)
Rat Snake	1	BL	2.86 (3)	–	–	–	King (2000)
Hognose Snake	1	BL	1.56 (3)	–	–	1.69 (3)	Iverson (2019)
Alligator (unstressed)	1	BL	–	1.51 (3)	–	–	Murray, Easter, Merchant, Cooper, and Crother (2013)
Alligator (stressed)	1	BL	–	0.63 (3)	–	–	Murray et al. (2013)

Note

Only statistically significant log–log slopes are reported here. Size measures are body mass (BM), body length (BL), or body volume (BV). Reproductive correlates are clutch size (CS), egg mass (EM), clutch volume (CV), or clutch mass (CM). For studies of multiple species, mean value appears above range (in parentheses). Expected slope appears in parentheses following reported slope.

## 5 CONCLUSIONS

On average each of the reproductive variables we examined related to shell length hypoallometrically in contrast to expectations that log–log slopes would be isometric. Clutch mass (modal slope = 2.5–2.8; predicted slope = 3.0) and pelvic aperture width (=0.80–0.90; predicted slope = 1.0) scaled most closely to isometry, while clutch size (1.7–2.0; 3.0), egg width (0.5; 1.0), and egg mass (1.1–1.3; 3.0) deviated significantly from isometry. As we have demonstrated, understanding variation in allometry is also complicated by the effects of variation among at least individuals, populations, years, clutch number, and possibly habitat, as well as sample size. Future studies should focus on increased sampling in order to tease apart the relative impacts of these and other life‐history factors on reproductive allometry within turtle species. Furthermore, we are aware of no similar meta‐analysis of allometric slopes for any other animal taxon.

In order to test our preliminary conclusions regarding persistent intraspecific hypoallometry, we strongly encourage field biologists to submit the reproductive data they have already recorded to allometric analyses and publish (or archive) those findings. Only by collecting such data will a real understanding of reproductive allometry in turtles (and other taxa) be possible. Correlation and regression analyses relating reproductive output to female body size, when presented without log–log transformations, are of little utility to comparative studies and do little to advance our understanding of how energy is invested in reproduction.

## CONFLICT OF INTEREST

None declared.

## AUTHOR CONTRIBUTIONS

All three authors jointly conceived the project and collected the data. JBI analyzed the data and led the writing of manuscript, although PVL and JL contributed substantially to manuscript preparation and revision. All three authors gave final approval for publication.

## Data Availability

All processed data for this study appear in Table 1. Raw data were unavailable for published works and not authorized for sharing by colleagues who provided summary allometric statistics.
